# Comparison of Clinical and Radiologic Outcome of Adolescent Idiopathic Scoliosis Treated with Hybrid Hook-Screw Instrumentation versus Universal Clamp System

**DOI:** 10.1155/2016/7639727

**Published:** 2016-10-30

**Authors:** Ebrahim Ghayem Hassankhani, Farzad Omidi-Kashani, Shahram Moradkhani, Golnaz Ghayem Hassankhani, Mohammad Taghi Shakeri

**Affiliations:** ^1^Orthopedic Research Center, Orthopedic Department, Imam Reza Hospital, Mashhad University of Medical Sciences, Mashhad, Iran; ^2^Orthopedic Department, Imam Reza Hospital, Mashhad University of Medical Sciences, Mashhad, Iran; ^3^Orthopedic Research Center, Ghaem Hospital, Mashhad University of Medical Sciences, Mashhad, Iran; ^4^Faculty of Medicine, Mashhad University of Medical Sciences, Mashhad, Iran

## Abstract

*Background.* In surgical treatment of adolescent idiopathic scoliosis (AIS), hybrid universal clamp system has been used by some authors. We aimed to compare the clinical and radiologic outcome of hybrid universal clamp with hybrid thoracic hook lumbar screw.* Methods.* A prospective study was performed on 56 consecutive patients with AIS, who had alternatively undergone a posterior spinal fusion and instrumentation with hybrid thoracic hook lumbar screw system (28 patients: group A) and hybrid universal clamp system (28 patients: group B) between June 2006 and January 2014 at Imam Reza University Hospital and had been followed up for more than two years. The comparison was according to radiographic changes, operative time, intraoperative blood loss, complications, and Scoliosis Research Society (SRS-22) outcome scores.* Results.* The preoperative mean curve Cobb angle was 58° ± 7° (42°–74°) in group A and 60° ± 9° (46°–75°) in group B. The mean final coronal curve correction was 60.4% and 75.5% in groups A and B, respectively (*P* = 0.001). Postoperative SRS outcome scores were also comparable.* Conclusion.* Universal clamp instrumentation had a significantly better curve correction and lower complication rate compared with hybrid thoracic hook lumbar screw. Both instrumentation methods had similar operative time, intraoperative blood loss, and postoperative SRS outcome scores.

## 1. Introduction

The most common systems nowadays used for surgical correction and instrumentation of adolescent idiopathic scoliosis (AIS) are multisegment fixation systems [1–3]. There are various types of posterior instrumentation systems for idiopathic scoliosis such as all hook, all pedicular screw, or hybrid thoracic hook-lumbar pedicular screw instrumentation [4–6]. These systems allow for deformity correction on the coronal, sagittal, and axial planes [2, 7]. The use of imaging techniques such as fluoroscopy, preoperative computed tomography, and navigation system has been recommended by several authors for insertion of pedicle screws to reduce the neurovascular complications related to malposition of pedicle screws [8, 9]. These techniques can not only improve proper pedicle screw insertion, but also increase the operating time and irradiation [4, 5, 10].

Recently a modified system comprised of soft sublaminar bands associated with metal jaws (clamps) has been proposed by some authors to provide more deformity correction and decrease the operating time, radiation exposure, and blood loss relative to the previous routine spinal implantation. These clamps strongly reduce the most deformed and deviated vertebrae located at the apical region of scoliosis while being attached to the longitudinal rods (hybrid universal clamp system). These soft sublaminar bands apparently decrease the neurovascular risks associated with pedicular screws or wires insertion, provide immediate stability, anchor around the strongest portion of the neural arch, apply less stress at any given point of the bony surface (relative to metal sublaminar hook or wire), and as a result may reduce the risk of cutout fractures during deformity reduction [11, 12]. The purpose of this study was to compare the clinical and radiologic outcome of the hybrid universal clamp with hybrid thoracic hook-lumbar screw instrumentation in the surgical treatment of adolescent idiopathic scoliosis.

## 2. Materials and Methods

After local institutional review board approval (record number: 930154), we carried out a prospective study on 56 consecutive AIS patients operated on by two surgeons between June 2006 and January 2014 at Imam Reza University Hospital. We included those patients with Lenke type 1, 2, or 3 who had undergone a single posterior approach with either hybrid thoracic hook and lumbar pedicular screw (group A: 28 cases) or hybrid universal clamp technique (group B: 28 patients). We excluded those cases needing two-stage approaches, with major lumbar curves, with congenital or neuromuscular scoliosis, and with scoliosis with underlying spinal cord disorders and those cases with less than 24 months of follow-up. Standing posterior-anterior and lateral and supine bending radiographs and magnetic resonance imaging (MRI) of the total spine were taken from all the cases. Preoperative flexibility (PF) was calculated as follows: [(preoperative standing Cobb angle − supine bending Cobb angle)/preoperative standing Cobb angle] × 100% [13].

**Figure 1:**
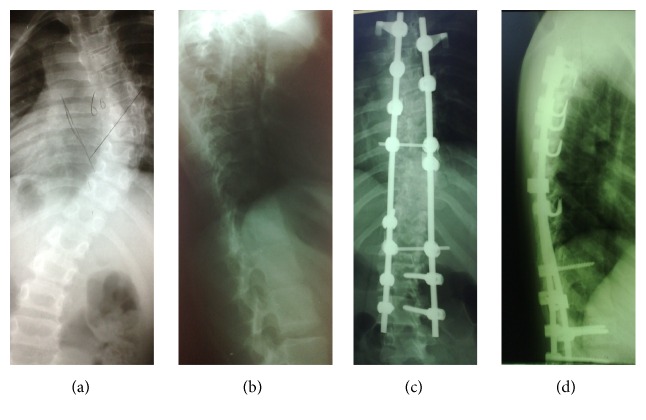
(a) and (b) A 17-year-old girl with AIS, with 2-year brace treatment. (c) and (d) After correction, PSF and instrumentation by hybrid thoracic hook lumbar screw technique.

**Figure 2:**
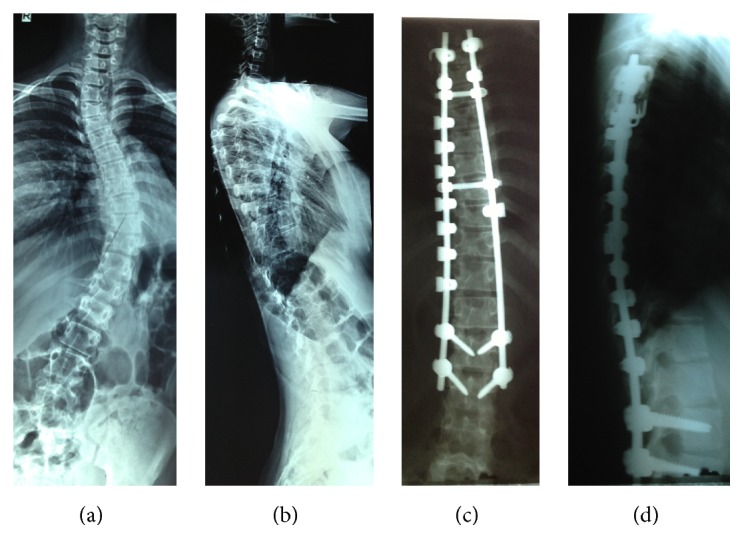
(a) and (b) A 16-year-old girl with AIS without brace treatment. (c) and (d) After correction, PSF and instrumentation by hybrid UC technique.

In group A, the classic derotation method was used for curve correction and then arthrodesis was performed by facetectomy, decortication, and bone graft (Figure 1). In this group, we aimed to use more screws relative to hooks, but in difficult situations or when the safety of the screw insertion was questionable, we inserted hooks, instead. In fact, all screw technique was too rare in our patients that were negligible. The construct used in group B (Figure 2) consisted of three parts. The proximal part consisted of hook claws (or hook-screw claws) on the two proximal vertebrae. In middle section, sublaminar universal clamp system was used in the concavity. One level was instrumented on the convex side. At the distal end, pedicle screws were used. Intraoperative fluoroscopic guidance was not used. Pedicle screws were placed using the free-hand technique. A frame was obtained with two precontoured 5.5 mm titanium rods united by three transverse connectors (we used the third connector in the apex due to resistance against deforming forces on the rods). The frame was secured to the proximal and distal end. The reduction of deformity was then begun in the center of curve to distal and proximal direction. When the frame was used to reduce the concavity of the thoracic curves, tension was applied to the UC system progressively. Distal screws were tightened at end of correction. Arthrodesis was performed by facetectomy, decortication, and bone graft. All patients had wake-up test during the operation.

After operation, we took standing posterior-anterior and lateral radiographs of the spine and calculated postoperative curve correction (POC) as follows: [(preoperative standing Cobb angle − postoperative standing Cobb angle)/preoperative standing Cobb angle] × 100% [13].

Comparison between pre- and postoperative curves was analyzed by paired-samples *t*-tests. We set statistical significance as a *P* value less than 0.05%. The SRS-22 questionnaire was used as our clinical outcomes' measurement tool. Reliability and validity of the Persian version of this questionnaire have already been confirmed [14].

## 3. Results

56 consecutive patients (42 female, 14 male) with AIS were included in the original study. None of the patients required anterior release and thoracoplasty. All of the patients with AIS (Lenke type 1, 2, or 3 curves) had posterior spinal fusion and instrumentation. 28 patients (20 female, 8 male) are treated with hybrid thoracic hook lumbar screw technique (group A) and 28 patients (22 female, 6 male) with hybrid universal clamp technique (group B). The mean follow-up period was 31.4 ± 5 months (range: 25–108 months).

In preoperative bending films, the PF of the main curve was 58 ± 8% in group A and 56 ± 11% in group B. The mean final coronal curve correction was 60.4% (from 58° ± 7° to 18.3° ± 5°) in group A and 75.5% (from 60° ± 9° to 15.7° ± 9°) in group B. This correction was highly significant, *P* < 0.0001 (Table 1). The mean improvements in sagittal curves were also depicted in Table 2.

There were no differences in the operative time (*P* = 0.25) and blood loss (*P* = 0.45). Postoperative SRS outcome scores were similar in both groups (group A: 94, and group B: 97, *P* = 0.19). There were 4 pedicle hook failures, 4 screws failures, and 3 superficial wound infections in group A and 2 pedicle hook failures in group B.

## 4. Discussion

The most important purpose in surgical treatment of idiopathic scoliosis is deformity correction on the coronal, sagittal, and axial planes with an effective fusion, fixation, and lowest possible rate of complications. There are various types of posterior instrumentation systems for idiopathic scoliosis such as all pedicular hooks, all pedicular screws, lumbar pedicular screws with thoracic hooks (hybrid), hybrid universal clamp, and sublaminar wiring techniques [4, 5, 15].

Many retrospective studies of patients with AIS treated with all-pedicle screw or lumbar pedicular screws with thoracic hooks (hybrid) instrumentation have suggested that conventional all-pedicle screw or lumbar pedicular screws with thoracic hooks (hybrid) constructs tend to worsen flatness of the thoracic spine in AIS [2, 4, 5, 7, 10, 16–18]. Recently Quan and Gibson concluded that in all-pedicle screw constructs the greater the coronal plane correction achieved, the greater the loss of thoracic kyphosis [16]. Vora et al. and Hicks et al. [10, 19] showed in presented series that sagittal balance was more satisfactorily corrected and preserved by hybrid universal clamp technique than by all-pedicle screw technique.

In this study we achieved better correction of coronal and sagittal plans in universal clamp than hybrid techniques. The mean coronal and sagittal curve correction was 75.5% and 80.8% in the universal clamp group while these parameters were 60.4% and 74.3% in hybrid group. To reduce the risk of neurovascular complications related to free-hand insertion of pedicle screws into the thoracic spine specially in all-pedicle screw technique [20, 21], the use of imaging techniques such as fluoroscopy and preoperative computed tomography is needed and recently neuronavigation has been recommended by several authors for safe placement of pedicle screws [8, 9]. These techniques inevitably increase operating time and irradiation exposure. Fluoroscopy is not needed for sublaminar anchorage hybrid universal clamp technique, which can consequently reduce the radiation exposure of the patient, surgeon, and other operating room professionals. In present study we had no significant difference in operation time of both series but irradiation exposure from fluoroscopy was high in all-pedicle screw technique.

There are many reports about the safety of hybrid universal clamp technique. Mazda et al. reported on a group of 75 AIS patients who received hybrid universal clamp technique. There were no complications related to the use of the hybrid universal clamp technique in their report [12].

In pedicle screw technique, screw-related complications may occur due to initial screw malposition or screw pull-out during correction maneuvers resulting in neurological, vascular, or visceral injury [22]. The rate of screw misplacement in the thoracic region has been reported as 5.7 to 50%, and the rate of neurovascular complications varies from 0 to 1% [23–27]. Other complications were infrequent and included pedicle fractures (0.24%), infections (1.9%), screw loosening (0.76%), and a single case of transient paraparesis [28, 29]. Abul-Kasim and Ohlin, in a consecutive series of 81 cases with AIS who had underwent scoliosis surgery, showed in one-third of patients minor screw loosening, 2 years after the intervention, evaluated by low dose CT [30]. We had 12 (5%) misplacements without neurovascular complication, 4 pedicle hook failures, and 4 screws failures in all-pedicle screw or lumbar pedicular screws with thoracic hooks (hybrid) technique but only 2 screws failures in the hybrid universal clamp technique.

Our study has some flaws. One of the most important defects of this study was the heterogeneity of the hybrid group. The ratio of screw to hook was varied but usually this ration was more than 80%, although we did not assess this matter exactly and statistically. We accept this as a flaw in our study and mentioned it in the text. It is recommended that a prospective study would be conducted on three patients groups: all-pedicle screw technique, hybrid hook-screw, and universal clamp, in the future.

## 5. Conclusion

Universal clamp instrumentation had a significantly better curve correction with lower complication rate compared with hybrid thoracic hook lumbar screw. Both instrumentation methods had similar operative time, intraoperative blood loss, and postoperative SRS outcome scores in the operative treatment of AIS.

## Figures and Tables

**Table 1:** Coronal preoperative, immediate, and final postoperative Cobb angle measurements and final correction in both groups.

Coronal Cobb angle measurements	Group A	Group B	Significance
Mean preoperative	58° (42–74)	60° (46–75)	NS
Mean immediate postoperative	24.5° (14–28)	15° (12.5–19)	*P* = 0.001
Mean final postoperative	28.1° (15–34)	17.4° (13–24)	*P* = 000.1
Mean final curve correction	60.4%	75.5%	*P* = 0.001

**Table 2:** Sagittal preoperative, immediate, and final postoperative Cobb angle measurements in both groups.

Mean sagittal Cobb angle	Curve	Group A	Group B	Significance
Preoperative	Thoracic	35.2° (3–56)	36.6° (0–68)	NS
	Thoracolumbar	13.8° (−8–22)	14.3° (−11–24)	NS
	Lumbar	−43.6° (−75 to −21)	−42.5° (−68 to −18)	NS

Immediate postoperative	Thoracic	26.6° (15–50)	30.8° (10–58)	NS
	Thoracolumbar	2.9° (−11–3)	2.1° (−13 to 15)	NS
	Lumbar	−42° (−70 to −10)	−40.6° (−61 to −20)	NS

Final postoperative	Thoracic	28.7° (11–55)	27.3° (12–54)	NS
	Thoracolumbar	5.2° (−14–5)	4.8° (−10–15)	NS
	Lumbar	−42° (−73 to −15)	−41.3° (−65 to −18)	NS
